# Psychometric Properties of the Serbian Version of the Arm, Shoulder, and Hand Disability Self-Assessment Questionnaire: Criterion Validity, Construct Validity, and Internal Consistency

**DOI:** 10.3390/jcm13195903

**Published:** 2024-10-03

**Authors:** Milos Vucetic, Vedrana Pavlovic, Suzana Milutinovic, Milan Stojicic, Natasa Milic, Dejan Aleksandric, Lazar Miceta, Bojan Petrovic, Aleksandar Matejic, Nina Rajovic, Vladislav Stanisic, Ana Tasic, Milena Dubravac, Srdjan Masic, Dejana Stanisavljevic

**Affiliations:** 1 Institute for Orthopedic Surgery “Banjica”, 11000 Belgrade, Serbia; dr.m.vucetic@gmail.com (M.V.); aleksandricdejan@gmail.com (D.A.); lmiceta@yahoo.com (L.M.); bprobin86@gmail.com (B.P.); matejicmladji@gmail.com (A.M.); 2 Faculty of Medicine, University of Belgrade, 11000 Belgrade, Serbia; smilutinovic@yahoo.com (S.M.); milan.stojicic@med.bg.ac.rs (M.S.); ana.gtasic@gmail.com (A.T.); 3 Institute for Medical Statistics and Informatics, Faculty of Medicine, University of Belgrade, 11000 Belgrade, Serbia; vedrana.pavlovic@med.bg.ac.rs (V.P.); natasa.milic@med.bg.ac.rs (N.M.); nina.rajovic@med.bg.ac.rs (N.R.); vladislavbstanisic@gmail.com (V.S.); 4 Clinic for Orthopedic Surgery and Traumatology, University Clinical Center of Serbia, 11000 Belgrade, Serbia; 5 Clinic for Burns, Plastic and Reconstructive Surgery, University Clinical Center of Serbia, 11000 Belgrade, Serbia; 6 Department of Internal Medicine, Division of Nephrology and Hypertension, Mayo Clinic, Rochester, MN 55902, USA; 7 Department for Primary Health Care and Public Health, Faculty of Medicine Foca, University of East Sarajevo, 71123 East Sarajevo, Bosnia and Herzegovina; srdjan.masic@ues.rs.ba

**Keywords:** the Disabilities of the Arm, Shoulder and Hand (DASH) questionnaire, confirmatory factor analysis, upper limb disability, internal consistency

## Abstract

**Background/Objectives**: The Disabilities of the Arm, Shoulder, and Hand (DASH) questionnaire is a widely employed self-report tool for assessing upper extremity function. The aim of this study was to assess the psychometric properties of the Serbian version of the DASH by determining its criterion and construct validity, as well as internal consistency. **Methods**: This cross-sectional study was conducted among patients with hand and wrist disabilities at the Institute for Orthopedics “Banjica”, Serbia. The psychometric properties of the Serbian version of the DASH were analyzed through an examination of its factorial structure and internal consistency. The DASH consists of 30 items, 24 of which assess function, 21 of which focus on physical function and three on social/role function. The remaining six items evaluate symptoms related to pain, tingling/numbness, weakness, and stiffness. **Results**: A total of 297 patients were included in the study. The mean age was 47.4 ± 16.8 years, with 50.5% males. Three models were assessed to determine the reliability and validity of the questionnaire across different domains. Model 1 examined a single-factor structure. In Model 2, the items were divided into two domains: Physical Function and Psychosocial/Symptoms. In Model 3, items were subdivided into three domains: Physical Function, Symptoms, and Psychosocial. All models demonstrated an excellent internal consistency with a Cronbach’s alpha > 0.9 for most domains. The values for the fit indices Tucker–Lewis index (TLI) and Comparative-Fit Index (CFI) were above their cut-off criteria of 0.9, while the Root Mean Square Error of Approximation (RMSEA) and Standardized Root Mean Square Residual (SRMR) were below the suggested value of 0.06, indicating an excellent level of models fit. Standardized factor loadings were statistically significant (*p* < 0.05). **Conclusions**: The present study provided the evidence for the appropriate metric properties of the Serbian version of the DASH. Results support both the unidimensional and multidimensional structures of the DASH.

## 1. Introduction

Disorders and injuries of the upper extremities are becoming increasingly significant due to their high economic burden on healthcare systems and detrimental impact on the quality of life for those affected [1]. A systematic review revealed that the prevalence of upper extremity disorders varied from 2% to 53% across different populations, with higher rates observed among students and employees [2]. Furthermore, the high prevalence rates and diverse outcomes resulting from upper extremity disorders affect daily activities, social well-being, and productivity [3].

To effectively assess the impact of upper extremity disorders on patients, self-reported questionnaires have emerged as valuable complements to objective clinical examinations [4]. Well-designed self-reported questionnaires provide crucial insight into patients’ subjective experiences, including symptom severity and functional limitations [5]. Given that symptom relief and disability reduction are the main reasons patients with musculoskeletal disorders of the upper extremities seek medical help, clinicians and researchers are increasingly incorporating the use of valid and reliable measurement instruments into practice [5,6].

Considering the complex nature of upper limb dysfunction, characterized by limitations in range of motion, muscle power, and pain perception, accurate assessment is essential for clinical management and research. To evaluate these impairments and their impact on daily life, a variety of assessment tools have been developed [7,8,9,10]. The Disabilities of the Arm, Shoulder, and Hand (DASH) questionnaire is a widely employed self-report tool for assessing upper extremity function [10]. Initially designed in English, the DASH questionnaire, has undergone extensive translation and cultural adaptation (in Arabic, Chinese, Dutch, French, German, Italian, Spanish, etc.) to facilitate its use in diverse populations with upper extremity disorders [11,12,13,14,15,16,17]. According to a recent systematic review [18], there are 37 different language versions of the DASH questionnaire, with evidence of validity presented in 50 articles.

The DASH questionnaire was first developed in 1996 as a unidimensional tool [10]. In 2001, Beaton et al. [19] reported that all 30 items of the DASH highly correlate with one another (loading on one factor in factor analysis), suggesting the unidimensionality of the scale (a single summed score). However, the structure of the DASH has since been the focus of further research aimed at cross-cultural adaptation and validation. Since the DASH questionnaire had not been validated for the Serbian population, the aim of this study was to assess the psychometric properties of the Serbian version of the DASH by determining its criterion and construct validity, as well as internal consistency.

## 2. Materials and Methods

**Figure 1:**
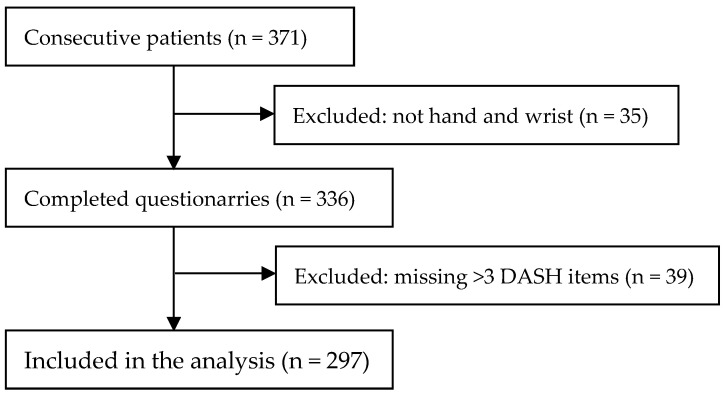
Flow chart of study participants.

This was a cross-sectional study conducted among patients with disabilities of hand and wrist (Figure 1). The study was performed at Institute for Orthopedics “Banjica” from January to May 2024. All study participants provided written informed consent for participation in the study. Participation was volunteer-based and was conducted with full preservation of data confidentiality, according to the principles of good research practice. Ethical approval was granted from the Ethical Review Board of the Institute for Orthopedics “Banjica” (reference number: i-113/7; date: 3 April 2023).

The research instrument was a self-reported questionnaire that consisted of four sections:

Socio-demographic information: The first part of the questionnaire included personal demographic information about the patients. Additionally, data regarding dominant hand and affected fingers were collected.

The Disabilities of the Arm, Shoulder, and Hand (DASH): The DASH questionnaire is a self-administered tool specifically created to assess physical function and symptoms in individuals with musculoskeletal problems affecting the upper extremities. The total score of the questionnaire is comprised of 30 individual components. Twenty-four items assess function, with 21 focusing on physical function and three on social/role function. The remaining six items evaluate symptoms: three are related to pain, one to tingling/numbness, one to weakness, and one to stiffness. Each item is scored on a 5-point Likert scale, where 1 represents no trouble or symptoms, and 5 signifies extreme difficulty (inability to perform) or severe symptoms. The DASH score is calculated subsequently, ranging from 0, showing an absence of disability, to 100, signifying the most severe level of disability. A higher score indicates a more significant level of disability. The DASH score was not estimated if there were more than three missing items [10].

Short-Form Health Survey (SF-12): The assessment of quality of life was conducted using the Serbian version of the 12-item Short-Form Health Survey (SF-12). The SF-12 questionnaire consists of 12 items, divided into two sets of six, used to generate a physical component summary score (PCS) and a mental component summary score (MCS). Higher scores on PCS indicate a better physical health-related quality of life, and higher scores on MCS indicate a better mental health-related quality of life. Responses to questions are expressed on an ordinal (always to never, excellent to poor) or dichotomous (yes/no) scale. In order to calculate PCS and MCS scores, a norm-based scoring algorithm was used, as recommended by Ware [20]. The regression weights and constants used in algorithm for both measures are derived from the general US population. The PCS and MCS scales are standardized with a mean of 50 and a standard deviation of 10 within this population. This standardization allows for meaningful comparisons between PCS and MCS scores, as well as straightforward interpretation relative to the general population’s score distribution. Specifically, scores above and below 50 indicate above- and below-average performance, respectively. Additionally, due to the standard deviation of 10, a one-point difference in scores corresponds to one-tenth of a standard deviation, offering a precise measure of variability.

Visual Analogue Scale (VAS): The visual analog scale (VAS), a commonly used assessment instrument with well-documented reliability and validity, was used for measuring the intensity of pain patients feel. This method enables patients’ pain levels to be measured across a continuum, from zero to an extreme amount. In this study, we asked patients to mark their pain level on a 10-cm horizontal line, labeling the left end as ‘no pain’ (0 cm) and the right end as ‘worst pain possible’ (10 cm).

### Statistical Analysis

Numerical data were presented as means or medians with corresponding measures of variability (standard deviations, 25th to 75th percentiles). Categorical variables were summarized by absolute numbers with percentages. The minimum sample size required for conducting factor analysis was determined based on the following recommended criteria: (1) a minimum of 150 participants for factor analysis, and (2) at least five to seven participants per item in the questionnaire. Since the DASH questionnaire consists of 30 items, the minimum suggested sample size for this study was 150 participants [21]. However, due to the prevalence of hand disabilities, the sample size was expanded to meet even stricter criteria. The psychometric properties of the Serbian version of the DASH were analyzed through the analysis of factorial structure and internal consistency (reliability). The internal consistency of the Serbian version of the DASH was assessed by using Cronbach’s alpha coefficient. Spearman’s correlation coefficients were calculated to explore the relationship between the DASH, VAS, and two SF 12 subscales. The construct validity of the Serbian version of the DASH was tested using confirmatory factor analysis (CFA). The absolute goodness-of-fit of the DASH models was evaluated using the Chi-square test (values that are <0.05 signify that a model may be a bad fit for the data). In addition, the Comparative-Fit Index (CFI), Tucker–Lewis index (TLI), Standardized Root Mean Square Residual (SRMR), and the Root Mean Square Error of Approximation (RMSEA) were used for model fit. Values of CFI and TLII above 0.90 were considered adequate, whereas SRMR and RMSEA values below 0.06 indicated an acceptable model fit. The reporting was performed in accordance with the recommendations for reporting the results of studies of instrument and scale development and testing [22]. All tests were two-tailed. In all analyses, the significance level was set at 0.05. Statistical analysis was done using Amos 21 (IBM SPSS Inc., Chicago, IL, USA, 2012) and IBM SPSS Statistics 25 software.

## 3. Results

A total of 297 patients with hand and wrist disabilities were included in the study. The mean age of the participants was 47.4 ± 16.8 years, and the gender distribution was almost equal, with 150 males (50.5%) and the remaining females. The mean DASH score was 40.4 (95% CI for mean: 37.4–43.3). The mean SF-12 subscale scores for PCS and MCS were 40.8 (95% CI for mean: 39.7–41.8) and 44.3 (95% CI for mean: 43.2–46.3), respectively. The detailed demographic and clinical characteristics of the study population are summarized in Table 1. Most common diagnoses were benign neoplasm of connective and other soft tissue, unspecified; abscess of tendon sheath, forearm; palmar fascial fibromatosis (Dupuytren) and carpal tunnel syndrome.

Participants’ responses to the DASH items are presented in Supplementary Table S1. For opening a tight or new jar, 24.5% reported no difficulty, while 16.3% were unable to do so. Writing was reported as having no difficulty by 49.7% of participants, but 14.8% found it impossible. Heavy household chores were manageable for 27.5% without difficulty, but 20.0% were unable to perform them. Similarly, 26.3% of patients were unable to perform gardening or yard work. Washing their backs and washing or blow-drying their hair were impossible for 19.0% and 15.6% of respondents, respectively. Recreational activities involving free arm movement were easy for 29.4% of patients, while they were impossible for the same number of participants (29.4%). Regarding social activity interference due to arm, shoulder, or hand problems, 11.1% of patients found it extremely interfering. Limitations in work or daily activities were reported by 21.6% of respondents, and 8.5% were experiencing extreme pain (Supplementary Table S1).

Supplementary Table S2 presents the frequency of patients’ responses to the DASH additional items (the DASH work module and the DASH sports/performing arts module). Among participants, 23.1% were unable to use their usual technique for work, and 23.8% could not perform their usual work due to arm, shoulder, or hand pain. Additionally, 27.6% of patients were unable to do their work as well as they would like, and 31.7% could not spend their usual amount of time doing it. One third (33.7%) of study participants were unable to use their usual technique for playing their instrument or sport, and 35.4% could not play their instrument due to pain. Furthermore, 39.8% were unable to play as well as they would like, and 36.4% could not spend their usual amount of time practicing or playing their instrument or sport (Supplementary Table S2).

The analysis of the internal consistency of the Serbian version of the DASH questionnaire is presented in Table 2. Three models were assessed to determine the reliability of the questionnaire across different domains. Model 1 examined the internal consistency of a single-factor structure comprising all 30 items of the DASH questionnaire. This model demonstrated an excellent internal consistency, with a Cronbach’s alpha of 0.966. In Model 2 the items were divided into two domains: Physical Function (21 items) and Psychosocial/Symptoms (nine items). The Physical Function domain as well as the Psychosocial/Symptoms domain both showed excellent internal consistency with a Cronbach’s alpha of 0.967 and 0.925, respectively. In Model 3 items were subdivided into three domains: Physical Function (21 items), Symptoms (six items), and Psychosocial (three items). The Physical Function and Symptoms domains showed excellent internal consistency with a Cronbach’s alpha of 0.967 and 0.923, respectively, while the Psychosocial domain indicated good internal consistency with a Cronbach’s alpha of 0.800 (Table 2).

**Figure 2:**
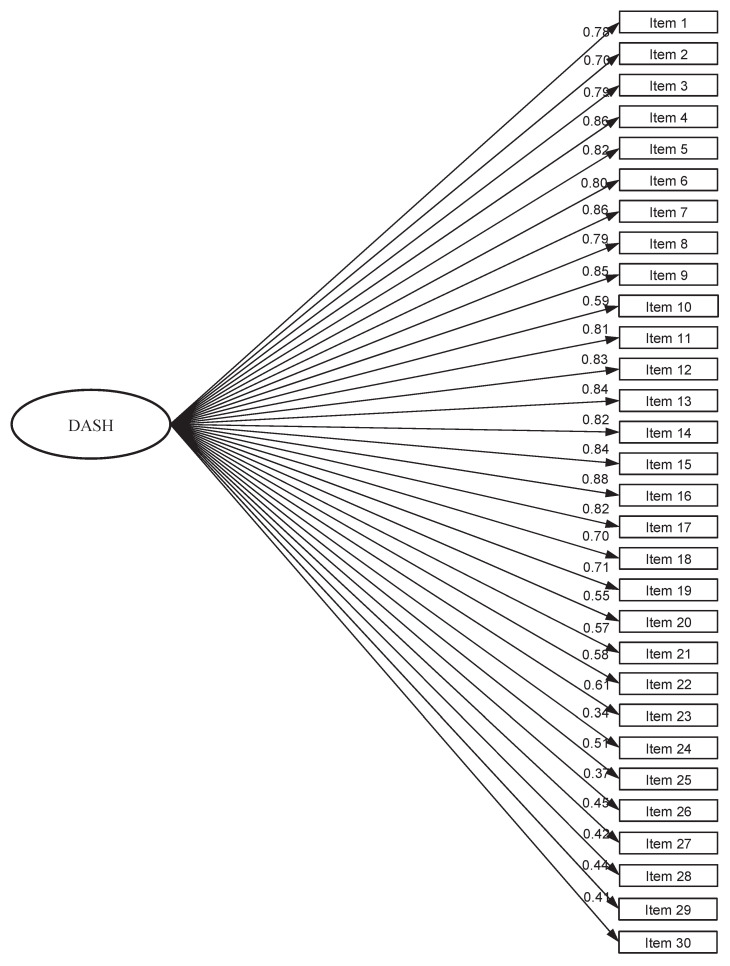
Single-factor model—Model 1.

The three different CFA models of the DASH questionnaire, with corresponding fit statistics, are presented in Table 3. Firstly, the CFA was performed to test the 30-item single-factor model fit (Figure 2, Table 3). The Chi-square test rejected the single-factor model as expected (χ^2^ = 606.98, *p* < 0.001), since this measure of fit is known to be sensitive to a large sample size. The RMSEA was 0.057 with a 90% CI of 0.050 to 0.063, and the SRMR was 0.039. The CFI and TLI values were 0.964 and 0.950, respectively, suggesting an excellent model fit. Factor loadings for the single-factor model of the DASH questionnaire are presented in Figure 2 and ranged from 0.34 to 0.88 (Figure 2).

**Figure 3:**
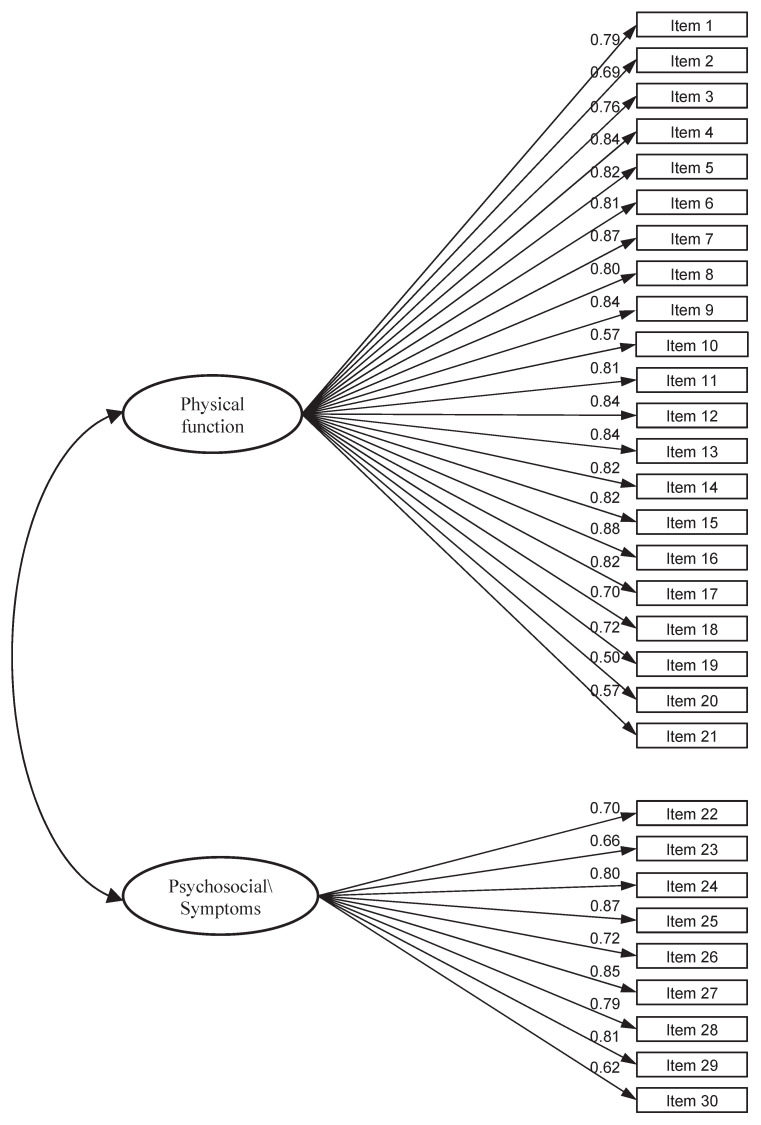
Bifactor model—Model 2.

Next, the CFA was run on the same sample to test the two-factor (Physical function and Psychosocial/Symptoms) model fit (Figure 3). The 30-item, two-factor structure of the DASH demonstrated an excellent fit of the data to the hypothesized model. The Chi-square test rejected the two-dimensional model (χ^2^ = 626.06, *p* < 0.001). In contrast, the RMSEA value of 0.053 (0.047–0.060) and SRMR of 0.046 were below the suggested value of ≤0.08. Values for fit indices TLI (0.956) and CFI (0.965) were above the cut-off of ≥0.95, indicating an excellent fit. All standardized factor loadings were statistically significant and ranged from 0.50 to 0.88 (Figure 3, Table 3).

**Figure 4:**
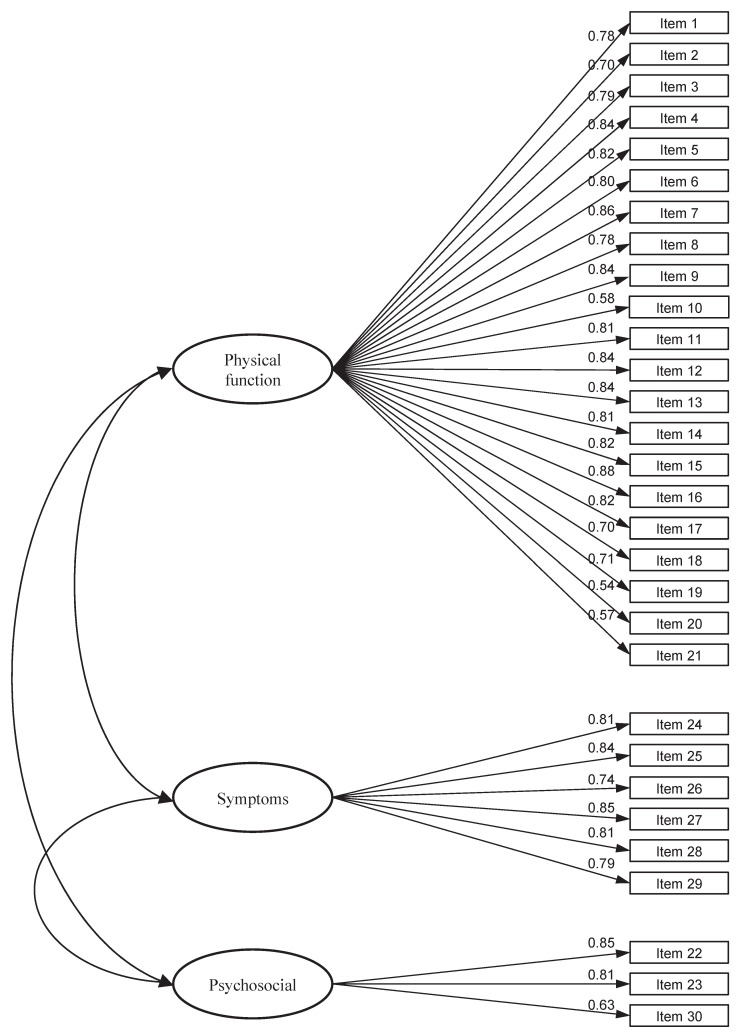
Three-factor model—Model 3.

The three-factor structure of the DASH questionnaire (Physical Function, Symptoms, and Psychosocial) has been validated, and the results are presented in Table 3. Model 3 also yielded a significant Chi-square test goodness-of-fit value, as expected (χ^2^ = 593.84, df = 331, *p* < 0.001). The values for the fit indices TLI (0.958) and CFI (0.968) were above their cut-off criteria, indicating an excellent level of model fit. The RMSEA value of 0.052 (0.045–0.058) and SRMR (0.040) were below the suggested value of 0.06, also suggesting adequate fit. Standardized factor loadings were statistically significant and ranged from 0.54 to 0.85 (Figure 4).

The correlations between DASH, SF 12 and VAS score are presented in Table 4. DASH was negatively correlated with both SF 12, subscale scores PCS and MCS (ρ = −0.564; *p* < 0.001 and ρ = −0.422; *p* < 0.00, respectively) and positively correlated with VAS (*p* < 0.001; ρ = 0.500) (Table 4).

## 4. Discussion

The present study provided the evidence for the appropriate metric properties of the Serbian version of the DASH. The various CFA models were used to clarify how the items of the Serbian version of the DASH relate to each other, and to explore if there were any subscale scores that should be used when scoring the questionnaire. This study suggests that the Serbian version of the DASH supports a unidimensional trait, although two- and three- dimensional models also yielded an excellent fit of the data.

Existing evidence suggests that the use of CFA should be prioritized for the assessment of the DASH’s structural validity [23]. It is recommended that the structural validity be assessed with CFA if there is prior evidence of dimensionality [18]. While Principal Component Analysis (PCA) is primarily employed for data reduction by attempting to account for all the variance within a dataset, EFA focuses on identifying the common variance among items to identify latent variables that were previously unknown. Conversely, CFA is utilized to assess whether empirical data align with a pre-specified factor structure derived from theory or prior research, making it particularly suited for validating the dimensionality of a questionnaire [24]. The selection of a specific factor analysis method is critical, as each technique is underpinned by distinct assumptions and addresses different research questions [25].

Thus far, Van Eck et al. [24] are the sole authors who have successfully carried out CFA using the Dutch version of the DASH, without first conducting Exploratory Factor Analysis (EFA). They conducted CFA to examine different models including a single factor model, a bifactor model and a correlated three-factor model. All models indicated a good model fit. Both one- and three-factor models showed high and statistically significant factor loadings (at least 0.70). However, the subscales Symptoms and Psychosocial had only three and two items, respectively, which could limit the construct’s theoretical domain coverage. Despite the fact that a two-factor model explained more variance, a one-factor model was preferred due to its simplicity. Based on their analysis, they concluded that the Dutch version of the DASH is indeed unidimensional. Our study results support both the unidimensional and multidimensional structure of the Serbian version of the DASH. The unidimensional model could be used when simplicity is needed; however, multidimensional models could be valuable when determining the contribution of subscale scores over and above the general factor.

In contrast, the Afrikaans version of the Western Cape DASH questionnaire presented internal consistency in both factors (subscales) and structural validity across two factors (physical function and psychosocial symptoms) [26]. The EFA showed that there are two factors, each with Eigenvalues greater than one, which together explain 55% and 7% of the variance. The CFA provided support for the two-factor structure of the Afrikaans for the Western Cape DASH questionnaire. The Cronbach’s alpha coefficients indicated strong internal consistency for both factors: factor 1 had a coefficient of 0.97 (95% CI: 0.96, 0.97), and factor 2 had a coefficient of 0.92 (95% CI: 0.90, 0.94). These findings align with our study results, which demonstrate excellent internal consistency for the bifactor model. Both factors—the Physical function and Psychosocial domain—exhibited excellent internal consistency, with Cronbach’s alphas of 0.967 and 0.925, respectively. However, the single-factor model of the Serbian version of the DASH also showed excellent internal consistency, with a Cronbach’s alpha of 0.966. Furthermore, in the three-factor model, the Physical function and Symptoms domains exhibited excellent internal consistency, with Cronbach’s alphas of 0.967 and 0.923, respectively, while the Psychosocial domain indicated good internal consistency, with a Cronbach’s alpha of 0.800.

Additional evidence for structural validity using CFA along with EFA exists for the Italian [14] and English (USA) [27] versions of the DASH. Franchignoni et al. investigated the factor structure of the Italian version of the DASH [14]. Following an exploratory analysis, the three-factor model demonstrated acceptable fit, although some items were misfitting. The one-factor model of the DASH was not supported due to suboptimal fit statistics. In their American version, Lehman et al. also examined a three-factor model, excluding items 20 and 21 due to their insufficient factor loadings [27]. Although the TLI and SRMR values suggested a good fit, the CFI and RMSEA did not. Additionally, they observed high correlations between the factors, exceeding 0.83.

CFA is therefore only conducted for four languages (Dutch, English (USA), Italian, and Afrikaans for the Western Cape) [14,24,26,27]. The assessment of structural validity using EFA was performed in the following language versions: French [28], German [13], Chinese [29], Igbo [30], Nepali [31], Persian [32], Japanese [33], Taiwanese [34], Yoruba [35], Portuguese (Brazil) [36], Kurdish [37], and Greek [38]. Rasch analysis has provided evidence of structural validity for the Dutch [39], Arabic [11], Finnish [40], and English (British) [41,42] language versions. Of the studies that investigated the factor structure of the DASH questionnaire, eight studies concluded that the questionnaire holds a single factor structure [11,24,29,32,33,34,39,41]. Two studies supported a two-factor structure [26,30], five a three-factor structure [13,14,27,36,43], one a four-factor structure [31], three a five-factor structure [28,37,38], and one a seven-factor structure [35].

To date, several reviews have been published focusing on the translation, cross-cultural adaptation, and validity of the DASH questionnaire [18,44,45,46]. Alotaibi et al. [34] provided a narrative synthesis on translation and cross-cultural adaptation of the DASH questionnaire and did not provide a quality assessment of the measurement properties following translation and cross-cultural adaptation. De Klerk et al. [45] investigated the evidence and quality of validity (including structural and cross-cultural validity) of the DASH questionnaire in developing country contexts, while Sigirtmac and Oksuz [46] investigated the quality of the cross-cultural adaptation (including structural and cross-cultural validity) of translated DASH questionnaires. De Klerk [18] in her scoping review aimed to identify the methods used for the psychometric evaluation of structural and cross-cultural validity of the DASH questionnaire. In most articles included in reviews, the process of the DASH translation and cross-cultural adaptation was outlined clearly and measurement properties, including validity, reliability, and responsiveness were reassessed. However, one of the main issues of the conducted studies is the inadequacy of the sample size [46]. According to the COSMIN checklist [47,48], an adequate sample size for a classical test theory is calculated as the number of items multiplied by seven, which in the DASH’s case, should have been at least 210 patients. Only a few publications had populations larger than 210 [46]. In our study, we included the recommended number of patients to meet the requirements of appropriate statistical analysis, which can be considered a strength of the study.

Furthermore, it is important to recognize that the qualitative process of translation and cross-cultural adaptation does not provide evidence of cross-cultural validity (which necessitates complex statistical analysis across multiple groups) [49]. To date, only one study has assessed cross-cultural validity (measurement invariance) of the DASH questionnaire after translation and cross-cultural adaptation, using Multiple Group Confirmatory Factor Analysis (MGCFA) for different languages [25]. Clinicians employing translated and cross-culturally adapted versions of the DASH questionnaire should be careful when interpreting results across different language versions in the absence of evidence supporting cross-cultural validity [18]. However, the MGCFA analysis conducted on a sample of 437 participants, consisting of 218 Afrikaans-speaking individuals from the Western Cape and 219 South African English-speaking individuals, demonstrated that the data support configural, metric, and scalar invariance models during the initial assessment of model fit. Further hypotheses testing comparing the nested models demonstrated that scalar invariance is upheld [26].

This study has some limitations. This was a single-center study with the inclusion of patients with variable levels of function of upper extremities. Although the presence of diverse diagnoses and treatments enhances the potential for generalizing findings, it can also be a limitation when more precise discrimination is required. The presence of heterogeneity emphasizes the necessity for additional assessment of the DASH in homogeneous patient groups, focusing on specific diseases and treatments. Despite these limitations, the response rate was sufficiently high and an adequate sample size was included. There was only a small number of missing values, from which total scores for all patients could still be calculated according to the DASH manual. CFA was used as the recommended analysis. Finally, future studies should assess validity in more detail, and other measurement properties of the DASH, such as test–retest reliability, responsiveness, and cross-cultural validation, should be performed.

Future directions. Hand disabilities are among the most prevalent types of disabilities in adults, significantly impacting daily activities in the short term and potentially leading to long-term effects, particularly for individuals whose work or home responsibilities require extensive use of their hands. The progress of recovery from a hand disability, regardless of the treatment approach, can be assessed through patient-completed questionnaires, such as the DASH administered, at regular intervals. However, a recent systematic review highlights a gap in the medical literature concerning the measurement properties of patient-reported outcome measures for individuals with hand fractures [50]. This underscores the need for further research to validate and use the DASH, which can be a crucial step for accurately measuring outcomes in both clinical studies and practice within the field of hand research.

## 5. Conclusions

In this study we assessed the psychometric properties of the Serbian version of the DASH questionnaire, by determining its criterion and construct validity, as well as internal consistency that were similar to other translated versions. The Serbian version of the DASH might be considered as comprehensive, reliable, and valid self-reported instrument to evaluate symptoms in patients with upper extremities disabilities.

## Figures and Tables

**Table 1:** Characteristics of study population.

Variables	*n* = 297
Age, mean ± sd	47.4 ± 16.8
Gender–male, *n* (%)	150 (50.5)
Dominant hand, *n* (%); *n* = 221	
Left	35 (11.8)
Right	186 (62.6)
DASH, mean (95% CI)	40.4 (37.4–43.3)
SF 12, mean (95% CI)	
PCS	40.8 (39.7–41.8)
MCS	44.3 (43.2–46.3)
VAS, median (25th–75th percentile)	4 (2–6)

**Table 2:** Internal consistency of the DASH questionnaire.

Model	Domains	N° Items	Cronbach’s Alpha	Internal Consistency
Model 1	Single factor	30	0.966	Excellent
Model 2	Physical function	21	0.967	Excellent
	Psychosocial/Symptoms	9	0.925	Excellent
Model 3	Physical function	21	0.967	Excellent
	Symptoms	6	0.923	Excellent
	Psychosocial	3	0.800	Good

**Table 3:** Fit statistics for DASH models.

	Chi-Squared Goodness of Fit	df	*p*	RMSEA (90% CI)	SRMR	CFI	TLI
Model 1	606.98	312	<0.001	0.057 (0.050–0.063)	0.039	0.964	0.950
Model 2	626.06	340	<0.001	0.053 (0.047–0.060)	0.046	0.965	0.956
Model 3	593.84	331	<0.001	0.052 (0.045–0.058)	0.040	0.968	0.958

df—degrees of freedom, *p*—*p* value, RMSEA—root mean square error of approximation, CFI—comparative fit index, TLI—Tucker-Lewis index, SRMR—standardized root mean square residual.

**Table 4:** Correlation coefficients (ρ) between DASH, SF 12 PCS, SF 12 MCS and VAS score.

		SF 12		VAS
		PCS	MCS	
DASH		−0.564 *	−0.422 *	0.500 *
SF 12	PCS	/	0.238 *	−0.548 *
	MCS	/	/	−0.465 *

DASH, Disabilities of the Arm, Shoulder, and Hand questionnaire; MCS, mental component summary score; PCS, physical component summary score; SF 12, Short-Form Health Survey; VAS, visual analogue scale. * *p* < 0.05.

## Data Availability

The datasets used and/or analyzed during the current study are available from the corresponding author on reasonable request.

## Supplementary Materials

The following supporting information can be downloaded at: https://www.mdpi.com/article/10.3390/jcm13195903/s1, Table S1: Participants’ frequency of responses to DASH items; Table S2: Participants’ frequency of responses to additional DASH items.
