# Supplementary material for: Psychometric Properties of the Serbian Version of the Arm, Shoulder, and Hand Disability Self-Assessment Questionnaire: Criterion Validity, Construct Validity, and Internal Consistency
Source: J Clin Med. 2024 Oct 3;13(19):5903. doi: 10.3390/jcm13195903 (PMC11478189; doi:10.3390/jcm13195903)
Supplement: Supplementary file 1 [file jcm-13-05903-s001.zip › jcm-3174448-supplementary.pdf]

Table S1. Participants' frequency of responses to DASH items

Items, n (%)					
Ability to do the following activities in the last week…	**No difficulty**	**Mild difficulty**	**Moderate difficulty**	**Severe difficulty**	**Unable**
Open a tight or new jar. n=294	72 (24.5)	61 (20.7)	64 (21.8)	49 (16.7)	48 (16.3)
Write. n=290	144 (49.7)	39 (13.4)	37 (12.8)	27 (9.3)	43 (14.8)
Turn a key. n=293	143 (48.8)	55 (18.8)	28 (9.6)	40 (13.7)	27 (9.2)
Prepare a meal. n=286	99 (34.6)	58 (20.3)	47 (16.4)	48 (16.8)	34 (11.9)
Push open a heavy door. n=287	85 (29.6)	71 (24.7)	42 (14.6)	49 (17.1)	40 (13.9)
Place an object on a shelf above your head. n=293	125 (42.7)	55 (18.8)	40 (13.7)	40 (13.7)	33 (11.3)
Do heavy household chores (e.g., wash walls, wash floors). n=295	81 (27.5)	60 (20.3)	54 (18.3)	41 (13.9)	59 (20.0)
Garden or do yard work. n=278	85 (30.6)	44 (15.8)	37 (13.3)	39 (14.0)	73 (26.3)
Make a bed. n=295	110 (37.3)	64 (21.7)	49 (16.6)	42 (14.2)	30 (10.2)
Carry a shopping bag or briefcase. n=295	97 (32.9)	75 (25.3)	49 (16.6)	44 (14.9)	30 (10.2)
Carry a heavy object (over 10 lbs). n=290	77 (26.6)	61 (21.0)	51 (17.6)	38 (13.1)	63 (21.7)
Change a lightbulb overhead. n=292	111 (38.0)	54 (18.5)	41 (14.0)	29 (9.9)	57 (19.5)
Wash or blow dry your hair. n=294	114 (38.8)	54 (18.4)	35 (11.9)	45 (15.3)	46 (15.6)
Wash your back. n=294	93 (31.6)	56 (19.0)	41 (13.9)	48 (16.3)	56 (19.0)

Put on a pullover sweater. n=294	113 (38.4)	62 (21.1)	44 (15.0)	42 (14.3)	33 (11.2)
Use a knife to cut food. n=294	106 (36.1)	57 (19.4)	44 (15.0)	38 (12.9)	49 (16.7)
Recreational activities which require little effort (e.g., cardplaying, knitting, etc.). n=290	113 (39.0)	54 (18.6)	42 (14.5)	28 (9.7)	53 (18.3)
Recreational activities in which you take some force or impact through your arm, shoulder or hand (e.g., golf, hammering, tennis, etc.). n=297	75 (26.0)	46 (16.0)	36 (12.5)	34 (11.8)	97 (33.7)
Recreational activities in which you move your arm freely (e.g., playing frisbee, badminton, etc.). n=279	82 (29.4)	38 (13.6)	45 (16.1)	32 (11.5)	82 (29.4)
Manage transportation needs (getting from one place to another). n=289	168 (58.1)	38 (13.1)	27 (9.3)	19 (6.6)	37 (12.8)
Sexual activities. n=281	141 (50.2)	47 (16.7)	35 (12.5)	21 (7.5)	37 (13.2)
	**Not at all**	**Slightly**	**Moderately**	**Quite a bit**	**Extremely**
During the past week, to what extent has your arm, shoulder or hand problem interfered with your normal social activities with family, friends, neighbours or groups? n=296	76 (25.7)	77 (26.0)	59 (19.9)	51 (17.2)	33 (11.1)
	**Not limited at all**	**Slightly limited**	**Moderately limited**	**Very limited**	**Unable**
During the past week, were you limited in your work or other regular daily activities as a result of your arm, shoulder or hand problem? n=296	59 (19.9)	55 (18.6)	50 (16.9)	68 (23.0)	64 (21.6)
The severity of the following symptoms in the last week…	**None**	**Mild**	**Moderate**	**Severe**	**Extreme**
Arm, shoulder or hand pain. n=294	44 (15.0)	83 (28.2)	61 (20.7)	81 (27.6)	25 (8.5)
Arm, shoulder or hand pain when you performed any specific activity. n=291	37 (12.7)	75 (25.8)	43 (14.8)	88 (30.2)	48 (16.5)

Tingling (pins and needles) in your arm, shoulder or hand. n=292	91 (31.2)	65 (22.3)	58 (19.9)	39 (13.4)	39 (13.4)
Weakness in your arm, shoulder or hand. n=294	67 (22.8)	74 (25.2)	39 (13.3)	66 (22.4)	48 (16.3)
Stiffness in your arm, shoulder or hand. n=296	86 (29.1)	72 (24.3)	46 (15.5)	52 (17.6)	40 (13.5)
During the past week, how much difficulty have you had sleeping because of the pain in your arm, shoulder or hand? n=297	**No difficulty**	**Mild difficulty**	**Moderate difficulty**	**Severe difficulty**	**So much difficulty that i can't sleep**
	126 (42.4)	59 (19.9)	54 (18.2)	40 (13.5)	18 (6.1)
I feel less capable, less confident or less useful because of my arm, shoulder or hand problem. n=294	**Strongly disagree**	**Disagree**	**Neither agree nor disagree**	**Agree**	**Strongly agree**
	74 (25.2)	44 (15.0)	51 (17.3)	77 (26.2)	48 (16.3)

Table S2. Participants' frequency of responses to DASH additional items

Items, n (%)	No difficulty	Mild difficulty	Moderate difficulty	Severe difficulty	Unable
**DASH WORK MODULE**					
Did you have any difficulty:					
Using your usual technique for your work? n=251	53 (21.1)	46 (18.3)	50 (19.9)	44 (17.5)	58 (23.1)
Doing your usual work because of arm, shoulder or hand pain? n=248	49 (19.8)	55 (22.2)	41 (16.5)	44 (17.7)	59 (23.8)
Doing your work as well as you would like? n=246	57 (23.2)	43 (17.5)	27 (11.0)	51 (20.7)	68 (27.6)
Spending your usual amount of time doing your work? n=246	55 (22.4)	36 (14.6)	27 (11.0)	50 (20.3)	78 (31.7)
**DASH SPORTS/PERFORMING ARTS MODULE**					
Did you have any difficulty:					
Using your usual technique for playing your instrument or sport? n=101	23 (22.8)	17 (16.8)	18 (17.8)	9 (8.9)	34 (33.7)
Playing your musical instrument or sport because of arm, shoulder or hand pain? n=99	21 (21.2)	18 (18.2)	14 (14.1)	11 (11.1)	35 (35.4)
Playing your musical instrument or sport as well as you would like? n=98	23 (23.5)	14 (14.3)	16 (16.3)	6 (6.1)	39 (39.8)
Spending your usual amount of time practising or playing your instrument or sport? n=99	25 (25.3)	15 (15.2)	12 (12.1)	11 (11.1)	36 (36.4)
